# The antimicrobial effect of Octenidine-dihydrochloride coated polymer tracheotomy tubes on Staphylococcus aureus and Pseudomonas aeruginosa colonisation

**DOI:** 10.1186/1471-2180-9-150

**Published:** 2009-07-25

**Authors:** Michaela Zumtobel, Ojan Assadian, Matthias Leonhard, Maria Stadler, Berit Schneider

**Affiliations:** 1 Department of Otorhinolaryngology, Head and Neck Surgery, Medical University of Vienna, Vienna, Austria; 2 Institute of Hygiene, Experimental Microbiology and Quality Research, Paracelsus Medical Private University, Salzburg, Austria; 3 Department of Hygiene and Medical Microbiology, Medical University of Vienna, Vienna, Austria

## Abstract

**Background:**

The surface of polymeric tracheotomy tubes is a favourable environment for biofilm formation and therefore represents a potential risk factor for the development of pneumonia after tracheotomy. The aim of this *in-vitro *study was to develop octenidine-dihydrochloride (OCT) coated polymer tracheotomy tubes and investigate any effects on *Staphylococcus (S.) aureus *and *Pseudomonas (P.) aeruginosa *colonization. Additionally the resistance of the OCT coating was tested using reprocessing procedures like brushing, rinsing and disinfection with glutaraldehyde

**Results:**

*Contamination with S. aureus*: Before any reprocessing, OCT coated tracheotomy tubes were colonized with 10^3 ^cfu/ml and uncoated tracheotomy tubes with 10^5 ^cfu/ml (P = 0.045). After reprocessing, no differences in bacterial concentration between modified and conventional tubes were observed.

*Contamination with P. aeruginosa*: Before reprocessing, OCT coated tubes were colonized with 10^6 ^cfu/ml and uncoated tubes with 10^7 ^cfu/ml (P = 0.006). After reprocessing, no significant differences were observed.

**Conclusion:**

OCT coating initially inhibits *S. aureus *and *P. aeruginosa *colonisation on tracheotomy tubes. This effect, however, vanishes quickly after reprocessing of the tubes due to poor adhesive properties of the antimicrobial compound. Despite the known antimicrobial effect of OCT, its use for antimicrobial coating of tracheotomy tubes is limited unless methods are developed to allow sustained attachment to the tube.

## Background

In comprehensive studies examining the aetiology of ventilator-associated pneumonia (VAP), *Staphylococcus (S.) aureus *and *Pseudomonas (P.) aeruginosa *have been found to be the most frequently isolated gram positive and gram negative organisms, respectively [1]. Nosocomial pneumonia in intensive care units (ICU) caused by *S. aureus *has increased steadily over the past two decades [2]. After only a few days of mechanical ventilation, the cuffed polymer endotracheal tube is coated with a thick bacterial biofilm which has to be regarded as a continuous source of bacterial colonization of the lower respiratory tract ultimately resulting in VAP [3]. Treatment of infections associated with medical devices is often frustrated by the inability of antibiotics to penetrate biofilms and the increasing resistance of microbes to antibiotics [4]. In unpublished studies, we have identified bacterial colonization of 10^6 ^colony forming units (cfu)/ml on cuffed tracheotomy tubes after 3 days of use.

Silver tracheotomy tubes with inherent antimicrobial properties previously used in patients with a permanent tracheostomy have been replaced with polymer tracheotomy tubes which have improved patient comfort. With the increasing use of un-cuffed polymer tracheotomy tubes, monitoring of biofilm formation has become important and regular reprocessing of the un-cuffed tracheotomy tube 1 to 2 times a day is usually recommended by the manufacturer in order to avoid infections.

In order to lengthen medical device usage and to improve patient safety with higher quality polymer tracheotomy tubes, coating with an antimicrobial agent has been suggested [5]. Octenidine-dihydrochloride (OCT) could represent a candidate compound since it has a broad-spectrum antimicrobial activity and low toxicity. Studies on resident skin flora have demonstrated the bactericidal and fungicidal efficiency of OCT [6].

The aim of this study was therefore to develop an OCT coated tracheotomy tube in cooperation with the Heimomed Company and to investigate the antimicrobial inhibitory effect of coated OCT on experimental biofilms formed by *S. aureus *and *P. aeruginosa in-vitro*. The OCT coating was then tested for resistance to the tube reprocessing procedures of brushing, rinsing and disinfection with glutaraldehyde.

## Results

Significant differences in bacterial contamination were observed between uncoated and OCT coated tracheotomy tubes (see "Additional file 1").

### Contamination with *S. aureus*

**Figure 1:**
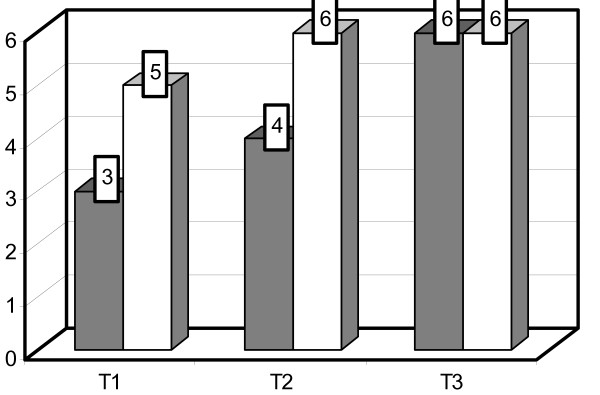
**Comparison of *S. aureus *colonization on OCT coated versus uncoated tracheostomy tubes**. Mean cfu concentration [log-] after standardized contamination with *S. aureus *before any reprocessing [T1], after 5 rounds of reprocessing [T2] and an additional 5 reprocessing procedures [T3]. OCT coated tracheostomy tubes are represented by gray bars, uncoated tubes by white bars.

Contamination *with S. aureus *showed the mean concentration of 10^3 ^cfu/ml on OCT coated tracheotomy tubes (group A) was significantly lower compared to uncoated tubes (10^5 ^cfu/ml; group B; P = 0.045). After five rounds of chemical reprocessing, a hundred fold difference between the colonization of both tube groups (group A = 10^4 ^cfu/ml; group B = 10^6 ^cfu/ml; P = 0.011) was observed. Following five further procedures of chemical and mechanical reprocessing, recontamination with *S. aureus *led to the similar colonization of both tube types (per Group: A+B = 10^6 ^cfu/ml; P = 0.115). These results are illustrated graphically in Figure 1.

### Contamination with *P. aeruginosa*

**Figure 2:**
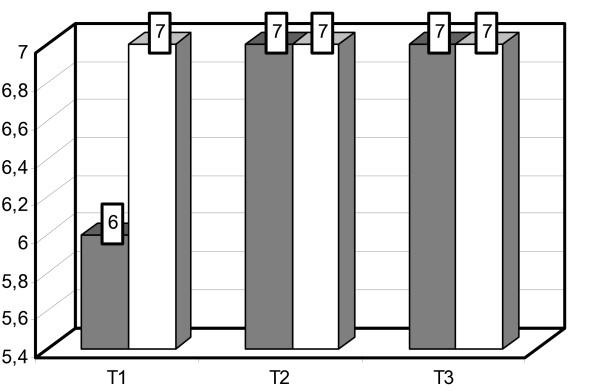
**Comparison of *P. aeruginosa *colonization on OCT coated versus uncoated tracheostomy tubes**. Mean cfu concentration [log] after standardized contamination with *P. aeruginosa *before any reprocessing [T1], after 5 rounds of reprocessing [T2] and an additional 5 reprocessing procedures [T3]. OCT coated tracheostomy tubes are represented by gray bars, uncoated tubes by white bars.

Prior to reprocessing, significant differences were seen between the mean concentration of *P. aeruginosa *colonization on OCT coated tracheotomy tubes (group C) of 10^6 ^cfu/ml and uncoated tracheotomy tubes (group D) of 10^7 ^cfu/ml (P = 0.006). After reprocessing, no statistical differences were observed (per group: C+D = 10^7^cfu/ml), P = 0.184 (Figure 2).

## Discussion

The goal of this study was to design an OCT coated polymer tracheotomy tube and to investigate antimicrobial inhibitory effects of the coating on *S. aureus *and *P. aeruginosa *colonization *in vitro*.

In current clinical practice, the use of polymer tracheotomy tubes leads to the early development of a thick biofilm followed by colonization of the lower respiratory tract as a potential risk factor for VAP, especially on cuffed tubes which are used for ventilation in ICU patients. Biofilm development starts after 6 hours and becomes abundant after 96 hours [7]. Different antiseptic agents embedded in or coated on polymer tracheotomy tubes have been proposed as an approach to reduce the bacterial burden and lower the risk of VAP development [8]. In this study, together with the manufacturer we developed OCT coated polymer tracheotomy tubes and investigated them in an experimental *in vitro *setting. The chemical, antimicrobial and toxicological properties of the bispyridine OCT has been described previously [9,10]. OCT is a potential non-alcoholic mucous skin and wound antiseptic, which destroys bacterial cells by interacting with their cell wall and intracellular components. Even at low concentrations (0.1% and below), OCT is considered bactericidal and fungicidal. In this study, a thousand-fold reduction in *S. aureus *colonization before reprocessing was achieved by OCT coating of the polymer tracheotomy surface. Although this result shows a favourable reduction required for antimicrobial medical devices [11], this activity vanished rapidly after tube reprocessing. Colonization of *P. aeruginosa *was inhibited less by the OCT coating than *S aureus *even before any reprocessing. In cuffed, single use tracheotomy tubes at the ICU, OCT coating might be of significant benefit because of the reduced *S. aureus *and *P. aeruginosa *bacterial burden. However, in the long-term use of un-cuffed polymer tracheotomy tubes, a benefit for the patient would not be expected due to the insufficient antimicrobial effects after daily reprocessing procedures as suggested by the manufacturer. These results are in contrast to recent studies that reported a sufficient reduction of *P. aeruginosa *on skin and dental plaques after application of OCT [12,13]. It is possible that the low concentrations of the OCT coating and poor adhesion to the tracheotomy tube polymer surface may explain the low antimicrobial effect. Superficial adhesion is thought to be rapidly eliminated by brushing and chemical reprocessing procedures. An alternative antimicrobial strategy might be to silver coat tracheostomy tubes which could prevent bacterial colonization more reliably and efficiently [14]. Although silver coating might be of clinical interest in the future, up to now its impact on VAP incidence has not been investigated thoroughly.

The results of this study have some limitations. We did not demonstrate the actual presence or examine the nature of the developed biofilms such as by using scanning electron microscopy of the colonized tracheotomy tubes in the presence or absence of OCT. However, the methods utilized are able to detect the presence or absence of bacterial colonisation even after a short time of 24 hours, which represents the initial step in any biofilm formation. Moreover, there is no marker suggesting a change in the pathogen metabolism after 24 hours. A study *in vivo *would be required to strengthen our results and some animal models suitable for investigation of tracheotomy tubes exist. However, in view of the discouraging results *in vitro*, we did not pursue further testing *in vivo *as we believe that based on our data, animal tests would be ethically unjustifiable. Finally, although VAP is associated with specific pathogens, bacterial biofilms have been described to be polymicrobic and the overall composition may greatly influence the bio-burden and infectious nature of the biofilm.

## Conclusion

In summary, OCT coating of tracheotomy tubes shows an antimicrobial effect and reduces colonization and biofilm formation on polymer tracheotomy tube surfaces. This effect diminishes quickly after reprocessing of the tubes. Therefore, despite the known antimicrobial effects, the use of OCT for antimicrobial coating of tracheotomy tubes seems to be ineffective in the absence of methods that allow sustained attachment of the antimicrobial compound to the tube.

## Methods

### Tube preparation

In order to prevent or delay formation of biofilms, a new polymer tracheotomy tube coated with OCT was designed in cooperation with Heimomed (Kerpen, Germany). The manufacturer coated its commercially available tracheotomy tubes with an adherent solution of OCT. These OCT coated tubes are currently not certified for *in vivo *use in patients and were prepared only for this study. For tracheotomy tube contamination, standardized test organisms of *S. aureus *(ATCC 6538) and *P. aeruginosa *(ATCC 9027) were used. For each pathogen, colonization on four tracheotomy tubes coated with OCT and four conventionally tracheotomy tubes was compared.

### Contamination

A suspension of 0.9% saline solution, 1% mucin and albumen imitated the adhesive properties of tracheal and bronchial mucous. An average of 10^6 ^cfu/ml was ascertained in this solution using a densitometer.

The suspension was filled into the inner lumina of all tubes. Excess fluid was removed after one hour of contamination at room temperature and the fully sealed tubes incubated for 24 h at 37°C. Segments (5 mm) were then excised from each tube and vortexed for 30 s in a neutralizing solution containing 5 ml of 0.9% saline and a combination of 3% saponin, 3% tween 80, 0.1% histidine and 0.1% cysteine for OCT inactivation. A series of 10-fold dilutions were made from each sample fluid and pipetted onto Mueller-Hinton/McConkey agar. Each dilution step was repeated in triplicate. After incubation at 37°C for 24 hours, the numbers of colonies were counted and analysed.

### Reprocessing procedures

*S. aureus *contaminated tubes were cleaned chemically with glutaraldehyde (2%) 5 times each and then re-contaminated. Manual brushing was added for the second reprocessing procedure. *P. aeruginosa *contaminated tubes were reprocessed mechanically and chemically 5 times between contamination procedures (Table 1).

### Statistical analysis

The number of pathogens was calculated as mean cfu ± standard deviation (SD) and presented in groups. The experiments were repeated in quadruplicate for 24 hours. A one-sided t-test was used to determine statistical significant differences. A p-value of < 0.05 was considered statistically significant.

## Authors' contributions

MZ performed the experiments, analysed and interpreted the data, as well as drafted and wrote the manuscript. ML participated in performing the experiments. MS participated in the study design and supervised the experiments. OA and BS had the idea for the study, participated in the study design and performed statistical analysis and analysed and interpreted the results. All authors have been involved in drafting the manuscript or revising it critically for important intellectual content and have read and approved the final manuscript.

## Supplementary Material

Additional file 1 **Overview of bacterial colonization on coated versus uncoated tracheotomy tubes**. The table illustrate the bacterial colonization on all 16 polymer tracheotomy tubes after contamination with *S. aureus *or *P. aeruginosa *at different experimental time points (T1, T2, and T3). Click here for file
